# Maternal Immunization with VP8* mRNA Vaccine Yields Superior Passive Transfer of Rotavirus-Neutralizing Antibodies to Foals

**DOI:** 10.3390/vaccines14010076

**Published:** 2026-01-09

**Authors:** Karin E. R. Borba, Rebecca M. Legere, Nathan M. Canaday, Jill W. Skrobarczyk, Zachary W. T. Arnold, Elena Cotton-Betteridge, Cristina Poveda, Michael F. Criscitiello, Angela I. Bordin, Luc R. Berghman, Jeroen B. K. Pollet, Noah D. Cohen

**Affiliations:** 1 Department of Large Animal Clinical Sciences, College of Veterinary Medicine & Biomedical Sciences, Texas A&M University, College Station, TX 77843, USA; karinborba@tamu.edu (K.E.R.B.); rlegere@tamu.edu (R.M.L.);; 2 6666 Ranch, Guthrie, TX 79236, USA; 3 Department of Poultry Science, College of Agriculture & Life Sciences, Texas A&M University, College Station, TX 77843, USA; jillpigs@tamu.edu (J.W.S.);; 4 Texas Children’s Hospital Center for Vaccine Development, Baylor College of Medicine, Houston, TX 77030, USA; cristina.poveda@bcm.edu; 5 Department of Veterinary Pathobiology, College of Veterinary Medicine & Biomedical Sciences, Texas A&M University, College Station, TX 77843, USA; mcriscitiello@cvm.tamu.edu

**Keywords:** mRNA, VP8*, rotavirus, vaccine, ELISA, foal

## Abstract

**Background**: Despite the availability of a killed whole-virus (KV) vaccine, diarrhea caused by equine rotavirus group A (ERVA) remains a significant health concern for foals in the United States. The vaccine is administered to pregnant mares, with foals protected by passive transfer of colostral antibodies. However, KV-induced immunity is only partially protective and maternal antibody levels in foals are often low and wane rapidly. To address these limitations, we developed a mRNA-based ERVA vaccine encoding the highly conserved VP8* protein to evaluate whether it can provide improved immune protection. **Methods**: Pregnant mares (n = 12 per group) were immunized either at months 8 and 10 of gestation with the VP8* mRNA or at months 8, 9, and 10 of gestation with the KV. Serum samples were collected from mares before and after immunization and from their foals at ages 1, 35, and 49 days. Serum samples were tested by indirect ELISA for VP8*-specific relative antibody concentrations and relative concentrations were compared for effects of study group and sample-time using linear mixed-effects regression. To detect functional antibodies against ERVA, a virus neutralization titer assay was performed to compare titers between mares vaccinated with the mRNA vaccine (and their foals) and unvaccinated control mares (and their foals). **Results**: Mares vaccinated with VP8* mRNA had significantly (*p* < 0.05) higher antibody concentrations after foaling than mares in the KV group, and foals of VP8* mRNA-vaccinated mares had significantly (*p* < 0.05) higher concentrations through age 49 days than foals in the KV group. In addition, the VP8* mRNA vaccine elicited higher titers of ERVA-neutralizing antibodies against both G3 and G14 strains. **Conclusions**: Longer-lasting, higher concentrations of virus-neutralizing antibodies might provide superior duration of immunity to ERVA in foals from mares vaccinated with VP8* mRNA.

## 1. Introduction

Rotaviruses of group A (RVAs) cause gastroenteritis in humans and animals worldwide [1]. Diarrhea is common among horse foals and equine RVA (ERVA) is the infectious agent most frequently detected in foals with diarrhea [2,3,4,5,6,7,8,9,10]. Infection with ERVA causes profuse diarrhea, dehydration, reduced suckling, lethargy, colic, and sometimes death [9,11,12,13,14]. Rotaviruses cause diarrhea primarily by infecting and replicating in the intestinal villus tip cells, ultimately lysing these cells, resulting in malabsorption, maldigestion, and hyperosmotic effects [15,16]. Other pathogenetic mechanisms contributing to intestinal dysfunction in ERVA infection include intestinal inflammation and ischemia caused by viral replication [9,10,15,16], activation of the enteric nervous system contributing to fluid and electrolyte secretion [16,17], and secretion of the rotaviral enterotoxin NSP4 which contributes to intestinal ileus [18,19]. Rotaviral diarrhea is particularly problematic for the horse industry because it often occurs in outbreaks with high morbidity at breeding farms [3,6,9,10,12,14,20,21].

Group A rotaviruses have 7 viral proteins (VPs), of which VP4 and VP7 are on the outer surface [10]. The VP4 comprises VP5* and VP8* [10]; VP8* is highly conserved among ERVA strains [10,20,22]. Variations in the VP4 and VP7 proteins are used to type ERVA: the VP7 is highly variable and is used to type RVA into strains referred to as G types, and variation in the VP4 is used to define P types of RVA. Most cases of ERVA foal diarrhea in the United States (U.S.) and other countries are caused by either the G3 or the G14 strains [10,12,20,21,22]. The most common P type of ERVA is P12 [10,22].

Protection against rotavirus in foals and other young animals (including human infants) is correlated with levels of antibodies against the virus, suggesting that vaccine-induced antibodies can protect against ERVA [7,9,10,12,22,23,24,25,26,27,28]. The vaccine currently available in the U.S. is conditionally licensed and lacks published evidence of significant efficacy to prevent diarrhea [29]. This vaccine is a killed whole virus (KV) product comprised of a single G3 strain of ERVA [10,29]. The vaccine is administered to mares at 8, 9, and 10 months of gestation to protect their foals by passive immunization. Despite the availability of this vaccine, diarrhea caused by ERVA remains an important health problem for foals in the U.S. and beyond [2,3,4,5,6,7,8,9,10,11,12,13]. This is at least in part because although the G3 vaccine induces antibodies that recognize G14 strains, the G14 cross-reacting antibodies occur at much lower levels of activity than those for G3 [22,30]. In central Kentucky, most foals that develop diarrhea attributed to ERVA are from vaccinated mares [7], and many foals that develop diarrhea are infected with G14 strains [9,22]. Many foals develop diarrhea caused by ERVA between the ages of 60 to 120 days, presumably due to waning maternal antibodies [7,9,14,22].

Collectively, these findings indicate that a more effective vaccine for ERVA is needed. Here, we describe a study comparing the immunogenicity of a VP8* mRNA vaccine with the commercially-available KV vaccine (Zoetis, Inc., Parsippany, NJ, USA). We hypothesized that the mRNA vaccine would provide higher concentrations of anti-VP8* antibodies in mares and their foals than the KV vaccine. Results indicated that 2 intramuscular doses of the mRNA vaccine elicited significantly higher anti-VP8 antibody responses in mares (that were passively transferred to their foals) compared to the KV vaccine. Moreover, the serum from mRNA vaccinated mares and their foals effectively neutralized a G3 and a G14 strain of ERVA, demonstrating that the in vivo–translated mRNA preserves the previously identified neutralizing epitopes of the VP8 antigen [10,20,22,28].

## 2. Materials and Methods

### 2.1. Virus and Cells

The ERVA strain used as a source of the viral sequence for design of mRNA construct and for neutralization assays was previously isolated from feces of 1– to 3-month old foals with diarrhea collected at farms in the Saratoga Springs, NY region, and confirmed by sequencing [28]; the VP8* sequence of the G3 isolate used in this study was identical to a previously reported ERV VP8 sequence publicly available in GenBank (ID: BAA02661.1). The G14 strain was generously provided by Dr. Feng Li, University of Kentucky and identified as RVA/Horse-tc/USA/KY-0316FCL/2021/G14P [12].

In vitro transfections were performed in Expi293 cells (ThermoFisher Scientific, Waltham, MA, USA, Cat # A14527), while the virus neutralization assay was performed in MA104 cells (African green monkey kidney epithelial cell line, ATCC^®^, Manassas, VA, USA).

### 2.2. Design of mRNA Construct

The mRNA construct was designed to encode the VP8* region of VP4 spike protein from the outer capsid P4 [12] VP4 protein of the equine rotavirus strain H-2 (GenBank accession for protein # AA70383.1; for mRNA sequence # L04638.1; https://www.ncbi.nlm.nih.gov/genbank/, (accessed on 15 September 2023)). The sequence extends from the N-terminus through the cleavage site, separating the VP8* and VP5* fragments. We aimed to include the 3 conserved neutralizing epitopes on the VP8* (aa 1–10, aa 55–66, and aa 223–234) [28]. The mRNA sequence was not codon-optimized for expression in equine tissues based on previous work from our laboratory [31]. This mRNA construct was in vitro transcribed (IVT) with a mammalian Kozak consensus sequence, base substitution with 5-methoxyuridine (5-moU), and polyadenylation by TriLink BioTechnologies, San Diego, CA, USA.

### 2.3. In Vitro mRNA Transfection

Expi293 cells were cultured in suspension according to the manufacturer’s recommendations. Briefly, 2.5 × 10^6^ cells were prepared in 25 mL of Expi293 expression media (ThermoFisher Scientific, Waltham, MA, USA, Cat # A1435102) in a vented 125-mL Erlenmeyer flask (ThermoFisher Scientific, Waltham, MA, USA, Cat # 4115-0125). After 24 h at 37 °C and 8% CO_2_ on an orbital shaker (125 rpm), 75 µg of mRNA per flask was prepared in 2.5 mL OptiMEM serum-free reagent (ThermoFisher Scientific, Waltham, MA, USA, Cat # 31985070), mixed gently, and incubated for 10 min at approximately 22 °C (i.e., room temperature). A volume of 112.5 µL of a commercially available transfection reagent (Lipofectamine MessengerMAX; ThermoFisher Scientific, Waltham, MA, USA, Cat # LMRNA008) was added into 2.5 mL OptiMEM serum-free reagent, mixed gently, and incubated for 10 min at approximately 22 °C. A total 2.5 mL of Opti-MEM containing 75 µg of mRNA were combined with 2.5 mL of Opti-MEM containing 112.5 µL of transfection reagent, mixed gently by inversion, and incubated for 48 h at 37 °C and 8% CO_2_ on an orbital shaker (125 rpm). Within 48 h after transfection, the cell culture suspension was collected and centrifuged at 4000× *g* for 20 min. The supernatants were harvested, filtered through a 0.22-µm syringe filter, and then concentrated using centrifugal filtration using an Amicon Ultra-15 centrifugal filter, 50 kDa MWCO (MilliporeSigma, Burlington, MA, USA, Cat # UFC9050) to a final volume of 500 µL from an original volume of 5 mL. Cells were washed once with PBS and lysed with radioimmunoprecipitation assay buffer and protease inhibitor cocktail − 100X (Sigma, St. Louis, MO, USA, Cat # P8340-1ML). The lysed cells and the concentrated supernatants were cryopreserved at −80 °C for future analyses.

### 2.4. Immunodetection of In Vitro Expressed VP8*

A Western immunoblot was performed to evaluate the expression and secretion of VP8* protein using concentrated supernatants and cell lysates of mRNA-transfected Expi293 cells. Samples were separated by SDS-PAGE (Mini-PROTEAN^®^ TGX™ Precast Gels, 4–20%, Bio-Rad Laboratories, Hercules, CA, USA, Cat # 4561096) and then transferred to a PVDF membrane (Bio-Rad Laboratories, Hercules, CA, USA, Cat # 1620177). Purified recombinant VP8* protein at a concentration of 0.25 ng/µL (synthesized by GenScript Biotech Corporation, Piscataway, NJ, USA) was used as a positive control. Membranes were blocked with 5% dried, non-fat milk in PBS-T (0.1%Tween-20 in PBS) for 2 h at approximately 22 °C in a rocker. The primary antibody was serum from a mare immunized against VP8* which was diluted 1:7500 and applied for a 2-h incubation. The membrane was then probed with a peroxidase-conjugated goat anti-horse IgG (H+L) (AffiniPure^®^, Jackson ImmunoResearch Laboratories, West Grove, PA, USA, Cat # 108-035-003) for a 2-h incubation at 1:3000 dilution. Protein bands were developed using Radiance Plus^®^ Femtogram HRP Substrate (Azure Biosystems, Dublin, CA, Cat # AC2103) and visualized using the Bio-Rad Chemidoc Touch imaging system.

### 2.5. Horses

**Figure 1:**
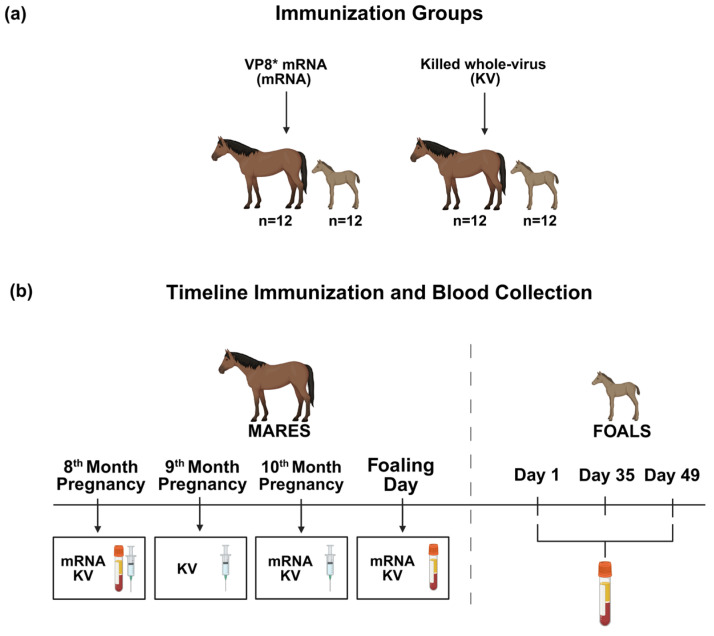
Study schematic and timeline of immunization and blood collection in pregnant mares and their foals. (**a**) Mares were divided into 2 groups of 12, 1 group receiving VP8* mRNA and 1 group receiving the KV. (**b**) Blood samples were collected from mares before immunization (8th month of pregnancy) and on the day of foaling. Blood samples were collected from foals at ages 1, 35, and 49 days. Figures were created in BioRender.

This study was approved by the Texas A&M University Institutional Animal Care and Use Committee and the Clinical Research Review Committee of the Texas A&M College of Veterinary Medicine & Biomedical Sciences (IACUC; Animal Use Protocol 2023-0202 CA). Immunized horses were from a large Quarter Horse breeding farm selected based on previous collaborations, excellent record keeping, expert and committed staff, and willingness to record data for the project. The farm had no history of recurrent incidence of rotaviral diarrhea, but broodmares were annually vaccinated with the commercially available killed whole virus (KV) rotaviral vaccine (Equine Rotavirus Vaccine, Zoetis, Inc., Parsippany, NJ, USA) at 8, 9, and 10 months of pregnancy. At the participating farm, 24 pregnant mares were randomly assigned using a blocked design by investigators at Texas A&M University using the psych package in R (version 4.4.3, R Foundation for Statistical Computing, Vienna, Austria) to either the VP8* mRNA vaccine (n = 12) or the commercially-available KV vaccine (n = 12) (Figure 1a). The sample size for this study was determined using the pwr.anova.test test in the pwr package of R statistical software by calculations based on power of 80% to detect differences between 2 groups, significance of 5%, and effect size of 0.5 based on ELISA data from preliminary results with this vaccine in non-pregnant horses and from another vaccine (targeting a conjugated polysaccharide) previously evaluated in pregnant mares by our laboratory [32]. Results indicated 12 horses per group were required (n = 24 horses total). For negative control sera for virus neutralization assays, blood was collected from 12 Quarter Horse mares of similar age as mares in the vaccine groups that had not been vaccinated against ERVA and their 12 foals. Mares and foals were monitored by veterinarians and veterinary technical staff at least twice daily.

### 2.6. Vaccine Formulations

The mRNA vaccine was comprised of 4 lots of VP8* mRNA, each containing 5 mg, formulated and prepared in lipid nanoparticles (LNPs) using a Genvoy ILM kit (Precision Nanosystems, Vancouver, BC, CA) following the manufacturer’s recommendations. The LNP kit consists of an ionizable lipid, DSPC, cholesterol, and PEG-lipid, with molar percentages of 50, 10, 37.5, and 2.5, respectively. VP8* mRNA LNPs were formulated at nitrogen to phosphate ratio of 4:1 using the NanoAssemblr Ignite instrument (Precision Nanosystems Inc., Vancouver, BC, Canada). To enhance LNP stability during freezing and reduce ethanol concentration, the LNPs were diluted in 1X PBS with 8% sucrose. The samples were subsequently concentrated to about 500 µg/mL using 10-kDa spin filter columns and filter sterilized before characterization. The RNA concentration in the LNP formulation of each lot was determined using the RiboGreen RNA Assay Kit (Invitrogen, Thermo Fisher Scientific, Waltham, MA, USA, Cat # R11490) with 1X TE buffer, with and without Triton X-100 detergent. The encapsulation efficiency of the RNA exceeded 85% for all batches/lots. The average LNP size and polydispersity index were assessed for each lot using dynamic light scattering (DLS) with a DynaPro Plate Reader II instrument (Wyatt Technologies, Santa Barbara, CA, USA). Based on DLS analysis, the VP8* LNPs exhibited sizes within the range of 80 to 100 nm (<12% polydispersity) for all lots. To evaluate the surface charge (pKa) of the LNPs, a 2-(p-toluidine)-6-naphthalene sulfonic acid (TNS) fluorescent assay was performed for each lot. The pKa of the LNPs fell within the range of 6.75 and 6.84, which aligns with the optimal pKa range of 6.6–6.9 for intramuscular (IM) vaccine delivery [33]. In a final step, the samples were diluted to 400 µg/mL using 1X PBS with 8% sucrose and aliquoted into sterile serum vials with a fill volume of 1.2 mL. The vaccines were shipped to the farm on dry ice and stored at −20 °C until use. One sample from each lot was set aside to monitor stability after thawing. The characterization results remained unchanged, and all biophysical parameters fell within the generally desired range for LNP mRNA vaccines.

The mRNA vaccine was administered to mares at months 8 and 10 of gestation (Figure 1b). The commercially available vaccine (Equine Rotavirus Vaccine; Zoetis Inc., Parsippany, NJ, USA) was administered IM to pregnant mares according to the manufacturer’s recommendations at the 8, 9, and 10 months of gestation. As noted above, mares included in the study had been immunized with this vaccine the preceding year. Vaccines were thawed and gently mixed immediately before administration. All IM immunizations were delivered to the neck in mares with a 3-mL syringe and 1.5-inch × 22-gauge needle.

### 2.7. Serum Sample Collection and ELISA Testing

Peripheral blood was collected by jugular venipuncture in clot tubes from mares before immunization at the 8th month of pregnancy and when their foals were between 12 and 24 h of age and from foals at ages 1 (i.e., at age 12 to 24 h when blood was collected to test for passive transfer of antibodies assessed by a semiquantitative immunoassay [SNAP Foal IgG Test, IDEXX, Portland, ME]), 35, and 49 days (Figure 1b). The serum was separated from the clotted blood, refrigerated, and shipped cooled overnight to the Equine Infectious Disease Laboratory at Texas A&M University. Serum samples were aliquoted and frozen at −80 °C until ELISA testing.

An in-house indirect ELISA was used to determine relative concentrations of VP8*-specific IgG antibodies in serum. The following steps were taken to ensure accuracy of the assay. We visually inspected the quality of test samples (e.g., no hemolysis) and test reagents used for the ELISA. Prior to testing samples from mares and foals, we optimized the ELISA steps to determine optimal volumes and times of washing steps, incubation times, and both quality and quantity (dilution) of reagents such as blocking buffer and secondary antibodies. We ensured there was no cross-reactivity with our high-affinity antibodies. Once we established a dilution for testing samples and an antigen concentration for coating plates, we demonstrated that the intra-assay coefficient of variation (CV) with positive control and test sera was <10% and that inter-assay CV was <15% for positive and negative control sera. On each test plate, we included serial dilutions of the positive control serum to confirm consistent dilutional effects were observed within and between assays. We included samples from all study groups on each plate and used plate-specific values of the OD for the positive control and blank to calculate OD ratios. For the ELISA, purified recombinant VP8* protein (synthesized by GenScript Biotech Corporation, Piscataway, NJ, USA) was used as the capture antigen. The positive control sample was serum from a commercial plasma donor horse hyperimmunized against ERVA (generously provided by Mg Biologics, Inc., Ames, IA, USA) and the negative control was pre-suckle serum collected from a foal born to a mare that had not been immunized against ERVA. Immunoassay plates (Nunc™ Maxisorp™ immunoassay plate; Thermo Fisher Scientific, Waltham, MA, USA; Cat # 44-2401-21) were coated with purified recombinant VP8* protein diluted in sensitization buffer (0.04 M PO4, pH 7.2) at a concentration of 2 µg/mL and then incubated overnight at 4 °C. Plates were washed with PBS with 0.05% Tween-20 and then blocked with 1% non-fat milk in PBS at 37 °C for 1 h. Serum samples collected from horses and foals in the study and positive and negative controls were added to the plate in duplicate (diluted 1:10,000; 100 µL/well). On each plate, samples from mares or foals from all study groups were included to ensure results were not biased by plate. Following washes, the peroxidase-conjugated anti-equine total IgG antibody at 1:5000 dilution (AffiniPure^®^ Goat Anti-Mouse IgG (H+L), Jackson ImmunoResearch Laboratories, West Grove, PA, USA, Cat # 108-035-003) was added to the plate, then incubated at 37 °C for 1 h. Plates were treated with SureBlue™ Reserve TMB Peroxidase Substrate (SeraCare Life Sciences, Milford, MA, USA, Cat # 5120-0081) and incubated for 5 min at approximately 22 °C in the dark. The reaction was stopped by adding sulfuric acid solution (0.18 M H2SO4), and ODs were determined at 450 nm using a microplate reader (Cytation™ 5 Cell Imaging, BioTek Instruments, Agilent Technologies, Winooski, VT, USA). Any values for which the OD values for duplicates varied by >15% were retested. An OD ratio was determined using the formula (Mean Sample OD − Mean Blank)/(Mean Positive Control − Mean Blank). The mean OD values for the positive control and blank were specific to the plate on which test samples were measured.

### 2.8. In Vitro Virus Neutralization

To detect functional antibodies against equine rotavirus, a virus neutralization titer assay was performed using MA-104 cell monolayers to determine the anti-ERVA titer, as previously described [28,34,35]. For the assay, 8 × 10^3^ TCID50 units of either a G3 strain of ERVA or a G14 strain of ERVA were incubated with serum collected from mares on the day of foaling (post-vaccination) and their foals at age 1 day from the mRNA-vaccinated group and unvaccinated control mares and their foals at these same time points. For the G3 strain, serum from all mares and foals were tested; for the G14 strain, samples from only 6 mares and foals in each group (mRNA vaccine or unvaccinated controls) were tested because of limited availability of serum. Our primary question of interest for this testing was whether the mRNA vaccine had induced virus-neutralizing antibodies in mares and foals relative to mares and foals in the unvaccinated control group. However, because we observed that relative concentrations of anti-VP8* antibodies were higher in the mRNA vaccinated mares and foals than the KV vaccinated mares and foals, we also compared VN titers in foals on day 1 in each of the 3 groups. Serum samples were serially diluted 2-fold, with dilution factors ranging from 1 to 512, and then incubated with the virus at 37 °C for 1 h. The virus and serum were then layered onto confluent MA-104 cell monolayers grown in a 96-well plate (Thermo Scientific™ Nunclon™ Delta surface 96-well plate, Thermo Fisher Scientific, Waltham, MA, USA, Cat # 167542). The following control wells were included in triplicate for each: (1) cells with media only, to verify that cells did not spontaneously develop cytopathic effects (CPE); (2) cells with media and serum to assess potential serum toxicity or other non-specific effects on the cells; and (3) cells with media, serum, and virus, to confirm the infectivity of the viral inoculum and provide a baseline for CPE. After 7 days of incubation at 37 °C in a humidified 5% CO_2_ atmosphere, the plates were examined for CPE. The complete absence of CPE was scored as negative for CPE and positive for neutralization. Antibody samples were run in duplicates, and the mean highest dilution of IgG where CPE was not observed was reported as the neutralization titer, calculated using the Reed method [36,37,38].

### 2.9. Data Analysis

All data were analyzed using descriptive and inferential methods with significance set at *p* < 0.05 using R statistical software (version 4.4.3). For descriptive analysis of the ELISA data, scatter plots of OD ratios by sample time, faceted by vaccine group, were generated. For inferential analysis of ELISA data, linear mixed-effects modeling was performed with the nlme package of R (version 4.4.3) to analyze the effects of sample time, vaccine group, and their interaction; post hoc analysis of pairwise differences between groups within time and times within groups was performed with Tukey’s method implemented using the emmeans package in R. Correlation of mare and foal VP8* OD ratios was performed using Spearman’s rank correlation test using the cor.test package in R with the Spearman option.

For the virus neutralization data from mares and foals, neutralizing titers were compared between the mRNA and control groups using a Wilcoxon rank-sum test. For the virus neutralization data comparing 3 groups from foals, titers were compared first using a Kruskal-Wallis test with post-hoc pairwise comparisons between groups (e.g., mRNA vs KV) tested using a Wilcoxon rank-sum test.

## 3. Results

### 3.1. Study Population

One mare was lost from the KV group for health reasons unrelated to vaccination; no data from this mare were included in analysis. One foal from the KV group and 1 from the mRNA group died from traumatic injuries within 24 h of foaling; no data from these foals were available for analysis. All other mares and foals remained free of signs of disease throughout the study, and no foals at the ranch were diagnosed with rotaviral diarrhea during the study period. No adverse effects were noted in vaccinated mares in either group.

### 3.2. In Vitro Translation

**Figure 2:**
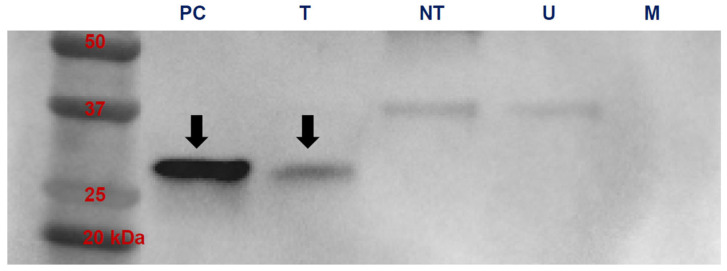
VP8* was expressed by Expi293 cells transfected with mRNA encoding the VP8* of ERVA. Western immunoblot of mRNA-transfected Expi293 culture supernatants (T) demonstrating assembly and secretion of VP8* protein; negative controls were supernatant of non-transfected cells (NT) treated with the lipofectamine transfection agent, supernatants from cells incubated without the lipofectamine transfection agent (U), and culture medium (M); positive control was purified VP8* (PC). A band was observed in the lanes for PC and T at the expected size for VP8* of 28 kDa (arrows), but not in the lanes of NT, U, or M controls.

Expi293 cells transfected with mRNA encoding VP8* expressed the protein in cell culture supernatants as demonstrated by Western immunoblotting (Figure 2). A band was seen at approximately 28 kDa for the positive control (purified recombinant VP8*) and from supernatants of Expi293 cells transfected with the VP8* mRNA construct used for our vaccine, but not in supernatants of non-transfected Expi292 cells with or without the transfection carrier (lipofectamine).

### 3.3. Anti-VP8* ELISA Results for Mares and Foals

**Figure 3:**
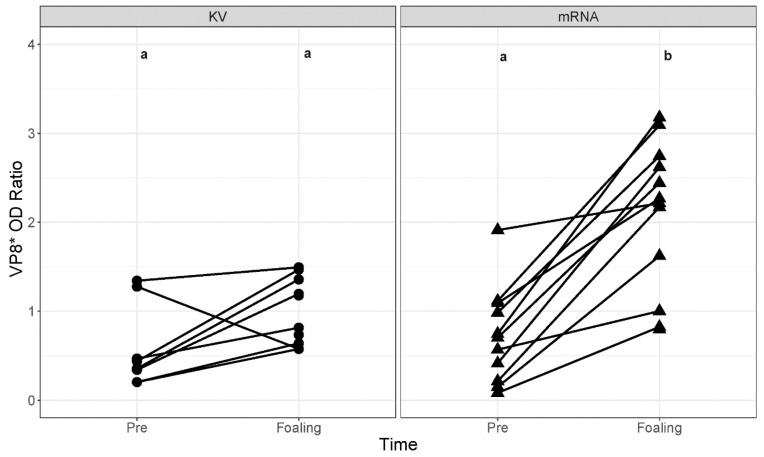
Relative concentrations of anti-VP8* antibodies were significantly higher after immunization in mares administered the VP8* mRNA vaccine. Scatterplots of anti-VP8* optical density (OD) ratios (OD of sample to OD of positive control) by timepoint (Pre = prior to immunization at 8th month of gestation; Foaling = sample collected on day of foaling) faceted by study group (KV = killed whole virus; mRNA = VP8* mRNA vaccine). Lines connect datapoints from each time for individual mares. Different letters (a vs b) indicate significant (*p* < 0.001) differences between groups and times.

Before immunization, serum VP8* OD ratios were not significantly (*p* = 0.7049) different between mares in the KV group (mean OD, 0.58; 95% CI, 0.14 to 1.01; *p* = 0.0339) and mares in the mRNA group (mean OD, 0.68; 95% CI, 0.30 to 1.07) (Figure 3). After immunization (at time of foaling), the OD ratio values tended to be increased but not significantly (*p* = 0.0815) for the KV group (mean OD, 1.00; 95% CI, 0.60 to 1.41); however, serum VP8* OD ratios were significantly (*p* < 0.0001) increased for the mRNA group (mean OD, 2.08; 95% CI, 1.71 to 2.45; *p* < 0.0001). Moreover, serum VP8* OD ratio values at foaling were significantly (*p* = 0.0036) higher for mares in the mRNA group than the KV group (Figure 3).

**Figure 4:**
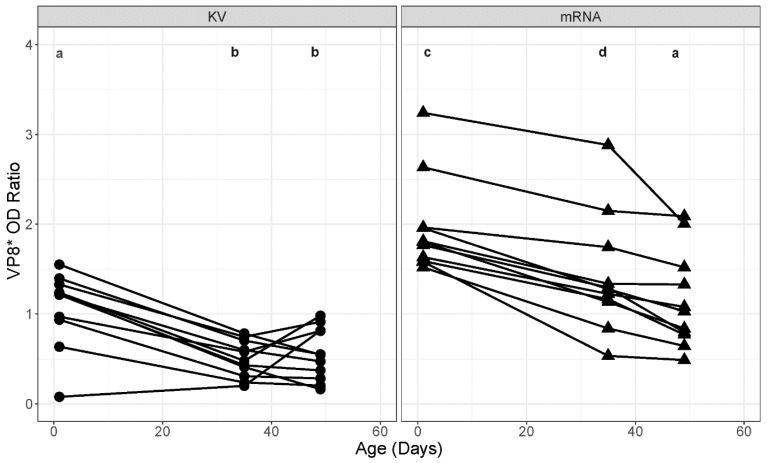
Relative concentrations of anti-VP8* antibodies were higher for foals born to mares vaccinated with the mRNA vaccine. Scatterplots of anti-VP8* optical density (OD) ratios (OD of sample to OD of positive control) by timepoint (Pre = prior to immunization at 8th month of gestation; Foaling = sample collected on day of foaling) faceted by study group (KV = killed whole virus; mRNA = VP8* mRNA vaccine). Lines connect datapoints for each time for individual mares. Different letters (a, b, c, or d) indicate significant (*p* < 0.001) differences between groups and times.

At age 1 day, the serum anti-VP8* OD ratio values were significantly (*p* < 0.0001) higher for foals in the mRNA group than for foals in the KV group (mean OD, 1.07; 95% CI, 0.78 to 1.36) (Figure 4). For foals in the KV group, VP8* OD ratio values were significantly (*p* < 0.0001 for both) lower at age 35 days (mean OD, 0.50; 95% CI, 0.21 to 0.79) and at age 49 days (mean OD, 0.56; 95% CI, 0.27 to 0.84) than at age 1 day, but values were not significantly different for the KV foals between ages 35 and 49 days (*p* = 0.9898).

For foals in the mRNA group, OD ratio values at ages 35 days (mean OD, 1.42; 95% CI, 1.13 to 1.70) and 45 days (mean OD, 1.14; 95% CI, 0.86 to 1.43) were significantly (*p* < 0.0001 for both comparisons) lower than at age 1 day, but values did not differ significantly (*p* = 0.0703) between ages 35 and 49 days for foals in the mRNA group (Figure 4). At age 35 days, VP8* OD ratio values were significantly (*p* = 0.0005) higher for foals in the mRNA group than foals in the KV group. At age 49 days, VP8* OD ratio values were significantly (*p* = 0.0495) higher for foals in the mRNA group than for foals in the KV group. Serum OD ratio values were not significantly (*p* = 0.9990) different between the mRNA group at age 49 days than for the KV group at age 1 day, the age when they were highest (Figure 4).

**Figure 5:**
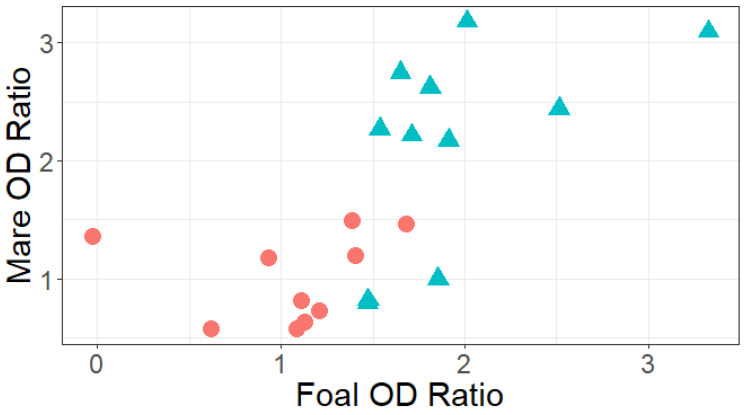
Correlation of anti-VP8* ELISA OD ratios of mares on the day of foaling and their foals at age 1 day. Mares were immunized either with the commercial killed virus vaccine (KV; coral circles; n = 11) or the VP8* mRNA vaccine (mRNA; teal triangles; n = 11). Values from foals were strongly (Spearman’s rho = 0.74) and significantly (*p* = 0.0002) correlated with values from their dams.

The relative antibody concentrations in mares and their foals were strongly (Spearman’s rho = 0.74) and significantly (*p* = 0.0002) correlated (Figure 5). These results further illustrate that values for the mRNA vaccinated mares and foals were higher than those for the KV group (Figure 5).

### 3.4. Virus Neutralization

**Figure 6:**
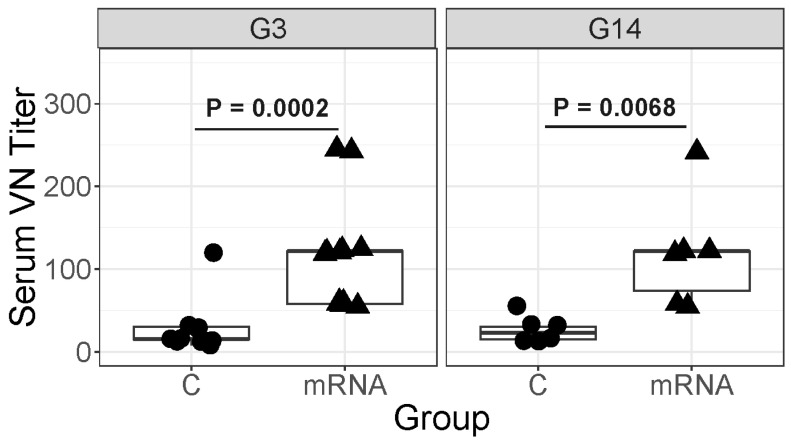
Serum ERVA neutralizing titers (VN) on the day of foaling for either mares vaccinated with VP8*mRNA or unvaccinated control (C) mares. Serum VN titers for the mRNA vaccinated mares were significantly higher than those of C mares for a G3 or a G14 strain of ERVA. Boxplots of serum ERVA neutralizing titers for a G3 strain of ERVA (left) or G14 strain (right). The horizontal lines at the top of the boxes represent the 75th percentile and the horizontal lines at the bottom of the boxes represent the 25th percentile; the 25th percentiles (bottom of boxes) overlap with the median (50th percentile) for C mares and the 75th percentile (top of boxes) overlaps with the median for the mRNA mares. The mRNA mares had significantly higher neutralizing titers than control mares for the G3 (*p* = 0.0002) and the G14 (*p* = 0.0068) strains.

On the day of foaling (Figure 6), anti-ERVA serum neutralizing titers for the G3 strain were significantly (*p* = 0.0002) higher for mares in the mRNA group (median titer, 121.5; range, 57.7 to 243.0) than for control mares (median titer, 15.2; range, 7.6 to 57.7), and serum neutralizing titers for the G14 strains were significantly (*p* = 0.0068) higher in mRNA-vaccinated mares (median titer, 121.5; range, 55.7 to 243.0) than control mares (median, 22.8; range, 15.2 to 57.7).

**Figure 7:**
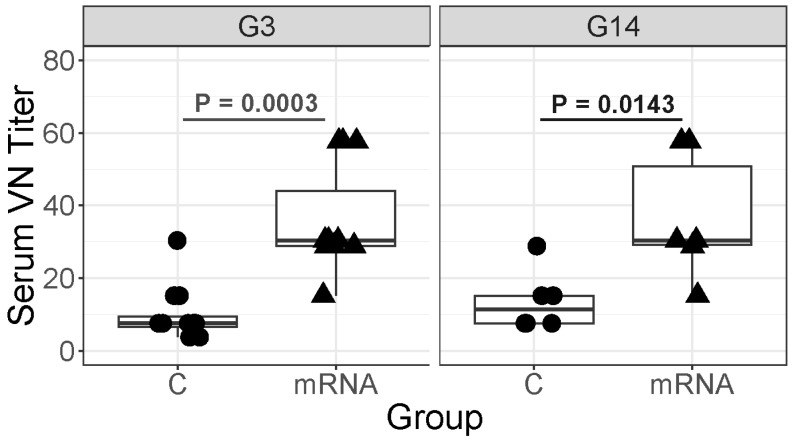
Serum ERVA neutralizing titers (VN) at age 1 day (after colostral absorption) for foals born either to mares vaccinated with VP8* mRNA (mRNA) or unvaccinated controls (C). Serum VN titers of foals of mRNA-vaccinated mares were significantly higher than those of foals born to C mares for a G3 and a G14 strain of ERVA. Boxplots of serum ERVA neutralizing titers for a G3 strain of ERVA (left) or G14 strain (right). The horizontal lines at the top of the boxes represent the 75th percentile and the horizontal lines at the bottom of the boxes represent the 25th percentile; the horizontal lines bisecting the boxes represent the median (50th percentile). The foals of mRNA vaccinated mares had significantly higher neutralizing titers than control mares for the G3 (*p* = 0.0003) or the G14 (*p* = 0.0143) strains.

At age 1 day (Figure 7), anti-ERVA serum neutralizing titers for the G3 strain were significantly (*p* = 0.0003) higher for foals in the mRNA group (median titer, 30.4; range, 15.2 to 57.7) than for foals in the control group (median titer, 7.6; range, 3.9 to 30.4) and anti-ERVA serum neutralizing titers for the G14 strain were significantly (*p* = 0.0143) higher in foals of mRNA-vaccinated mares (median titer, 30.4; range, 15.2 to 57.7) than control foals (median titer, 11.4; range, 7.8 to 28.8).

**Figure 8:**
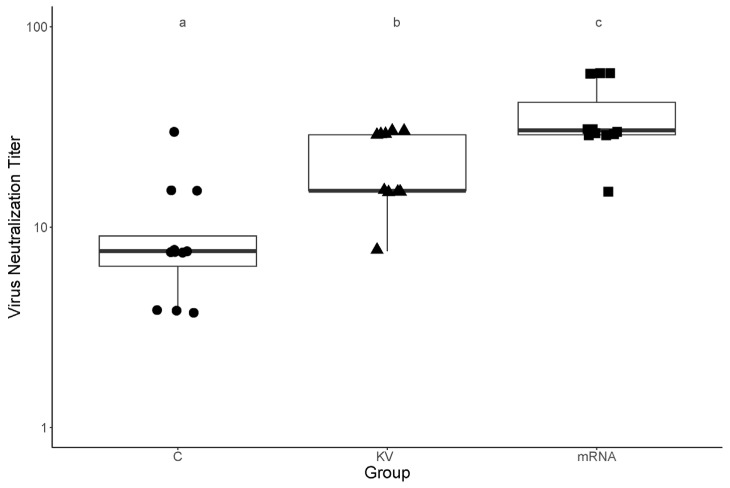
Serum virus neutralizing (VN) titers of foals at age 1 day for a G3 strain of ERVA. At age 1 day, serum VN titers were significantly (*p* < 0.05) higher for foals from mares vaccinated with VP8* mRNA vaccine (mRNA; n = 11) than for foals from either unvaccinated control mares (C; n = 11) or for foals from mares vaccinated with a killed whole virus vaccine (KV; n = 12). Boxplots of serum ERVA neutralizing titers. The thicker horizontal lines of the boxes represent the median. The bottoms and tops of the boxes represent the 25th and 75th percentiles of the data, and vertical lines extending from the bottoms or tops of the boxes extend to a multiple of 1.75 from the upper and lower quartiles of the data, respectively. Different letters (a, b, c) indicate significant (*p* < 0.05) differences between groups.

When comparing the serum neutralizing titers for the G3 strain of foals in the mRNA, KV, and control groups, anti-ERVA serum neutralizing titers differed significantly among the 3 groups (*p* = 0.0001; Figure 8). Considering pairwise comparisons between groups, serum ERVA neutralizing titers were significantly higher for foals in the mRNA group (median titer, 30.4; range, 15.2 to 57.7) than for foals either in the control group (*p* = 0.0003; median titer, 7.6; range, 3.9 to 30.4) or foals in the KV group (*p* = 0.0159; median, 15.2; range, 7.6 to 57.7). Titers for the KV group were significantly (*p* = 0.0038) higher than those of the control foals.

## 4. Discussion

Our results indicate that 2 doses of VP8* mRNA vaccine induced higher relative concentrations of anti-ERVA antibodies in mares that were passively transferred to their foals than 3 doses of the commercial KV. Because the VP8* is highly conserved in ERVA [10,20,22,28], it is expected that anti-VP8* antibodies will recognize multiple strains of ERVA, including the most prevalent strains (viz., G3 and G14 strains). Relative concentration of anti-VP8* antibodies in mare serum was correlated with that in their foals after absorption of passively transferred antibodies, and the higher relative concentration of anti-VP8* antibodies in mares resulted in higher and longer-lasting anti-ERVA antibody concentrations in their foals. Indeed, relative concentration of anti-VP8* antibodies in foals of the mRNA group at age 49 days was comparable to that at age 1 day for foals in the KV group (Figure 4), when activity levels were highest due to colostral absorption. Moreover, antibodies observed in mares and foals were able to mediate viral neutralization of both a G3 and G14 strain of ERVA, indicating that they might be functional in vivo to protect foals against rotaviral diarrhea.

None of the mares included in this study had adverse reactions to immunizations. The VP8* mRNA vaccine was administered to mares at months 8 and 10 of gestation, whereas the commercially available vaccine was administered at months 8, 9, and 10 of gestation (per the manufacturer’s recommendation). Administering only 2 injections rather than 3 injections has practical and financial advantages, including reduced handling (with attendant costs and chances of injury) and reduced risk of vaccine reactions.

This study had limitations. The vaccinated mares and their foals were from a single farm during a single year. Larger scale evaluation of the mRNA vaccine at multiple farms and in multiple years is warranted. The vaccinated mares in both groups (KV and mRNA) had all been previously immunized with the KV vaccine in preceding years. The importance of this limitation is somewhat diminished by unpublished data from preliminary safety testing we conducted with the VP8* mRNA vaccine in naïve mares indicating that these mares responded robustly to the mRNA vaccine despite no prior exposure. Moreover, it is probable that if the mRNA vaccine were used in the field that many mares would have been vaccinated previously against ERVA with the KV vaccine. We did not have an unvaccinated control group because the participating ranch declined to leave any mares unvaccinated against ERVA. This limitation is diminished by the fact that the study was self-controlled: each mare served as her own control, and the KV group was considered the control group for standard of care. The principal value of an unvaccinated control group would have been to confirm that the rising antibody concentrations after immunization of mares was not attributable to natural exposure to equine rotavirus. While this possibility cannot be excluded, the observation that no foals developed rotaviral diarrhea at the ranch during the study and prior evidence of immunogenicity of the KV vaccine [29] make it likely that the observed rising anti-VP8* antibody concentrations were the result of immunization. More importantly, the randomization of vaccine groups makes it highly improbable that the difference in relative antibody concentrations was attributable to differential natural exposure to ERVA in the mRNA group.

We only monitored immune responses to VP8*. Other antigens (e.g., VP7) in the KV vaccine might have induced higher relative concentrations of other antigens including antibodies that mediated virus neutralizing activity in horses that was not assessed by testing relative concentrations of anti-VP8* antibodies or anti-VP8* neutralizing activity in mRNA-vaccinated mares and their foals. We monitored foals until 49 days of age to assess the decay of passively transferred maternal antibodies, as rotavirus is most prevalent in young foals. However, results from central Kentucky, USA indicate that prevalence is highest at age 60 to 120 days [7,9]. Thus, longer duration of follow-up would have been informative and should be used in future studies. Nevertheless, it was important to monitor foals during the first 49 days to assess the decay of passively transferred maternal antibodies, and our results demonstrated that the mRNA vaccine provided higher antibody levels for a longer duration than the KV vaccine. We lack data regarding antibody concentrations or virus-neutralizing activity in mare’s milk. While foals absorb antibodies in colostrum into their systemic circulation during the first 24 h after birth, antibodies in milk could serve to neutralize ERVA in the gastrointestinal tract. Thus, monitoring antibody concentrations and virus neutralizing activity in milk samples would be worthwhile for future studies. We only compared virus neutralizing activity to the G3 and G14 strains in the mRNA-vaccinated mares and foals and unvaccinated control mares and foals because our primary aim for the virus neutralization assays was to demonstrate that the mRNA vaccine induced virus-neutralizing antibodies. Because our primary aim was to determine whether the mRNA vaccine induced higher anti-VP8* antibody concentrations than the KV, comparison of virus neutralization activity between the mRNA and KV groups would have been valuable. This limitation is mitigated by the fact that we demonstrated that virus-neutralizing titers for the G3 strain were significantly higher from foals immunized with the VP8* mRNA vaccine than the KV vaccine. Because limited serum was available as a result of optimizing our assays, we were only able to test serum from 6 mares and their foals in each of the mRNA vaccine and control groups. Our virus neutralization data were limited to a single strain each of G3 and G14. Although VP8* is highly conserved among strains such that our vaccine would be expected to induce neutralizing antibodies to many strains, more robust screening of broadly neutralizing capacity of antibodies induced by our VP8* mRNA vaccine is warranted. Last, we lack in vivo efficacy data; however, we observed that serum from both VP8* mRNA-vaccinated mares and their foals neutralized the virus, and viral neutralizing capacity has been correlated with efficacy in people and other animals [23,39]. Nevertheless, it is imperative that clinical efficacy of the VP8* mRNA vaccine to protect foals against rotaviral diarrhea be evaluated in vivo.

In summary, the VP8* mRNA vaccine appears to be an immunogenic approach that merits further investigation for its efficacy to protect foals against ERVA infection. Modifications to the vaccine that enhance immunogenicity might induce even higher anti-ERVA antibody concentrations and virus neutralizing activity levels in mares and their foals.

## 5. Conclusions

Our study demonstrated that mares and their foals vaccinated with a VP8* mRNA construct in LNPs had higher serum concentrations of anti-VP8* antibodies than mares vaccinated with the KV vaccine and their foals, and that mRNA vaccination elicited virus-neutralizing antibodies. Furthermore, antibody responses against ERVA VP8* remained elevated for a longer duration in foals from mares that received 2 doses of the VP8* mRNA vaccine than those that received 3 doses of the commercial KV vaccine. These findings suggest that the VP8* mRNA vaccine elicits a more robust and durable antibody response targeting the key neutralizing epitopes, supporting its potential as a superior alternative to traditional vaccines and warranting further investigation. Further evaluation of VP8* mRNA vaccines to protect foals against ERVA diarrhea is warranted.

## Data Availability

The raw data supporting the conclusions of this article will be available upon request from the corresponding author. The data are not publicly available due to privacy restrictions.

## Abbreviations

The following abbreviations are used in this manuscript:

ERVA	Equine rotavirus group A
mRNA	Messenger RNA
ELISA	Enzyme-linked immunosorbent assay
KV	Killed whole virus vaccine
RVA	Rotavirus group A
VP	Viral protein
LNP	Lipid nanoparticles
IM	Intramuscular
CV	Coefficient of variation
OD	Optical density
CPE	Cytopathic effect
IgG	Immunoglobulin G
